# Dental Diplomas in the State of New York

**Published:** 1882-07

**Authors:** 


### CORRESPONDENCE.

### DENTAL DIPLOMAS IN THE STATE OF NEW YORK.

To the Editor of DENTAL REGISTER:

This subject has been at some other time very mildly discussed. How men of good common sense can look on and see the tendency of the workings of such an affair as the New York society has been carrying on in the diploma business, and not blush for the mischief being done, is hard to determine. It is all wrong in principle. There is no need of such degrees. They are misleading the public and have a tendency to cheapen the value of the real merit of a degree. They are an insult to our schools of instruction.

Now, I positively know, from conversations with prominent members, that there is a revulsion of feeling in this matter. At Boston, during the meeting of the American Dental Association, this subject was brought up and discussed, in a lively manner, at the dinner table, and at that time I personally told a gentleman (a present member of the New York society and one who had just been elected to one of the most important offices) that it was a disgrace to allow this matter to go on; he replied that he knew it and felt it as much as I did, and he said: "I have determined to *beard* it at the next meeting, for I must acknowledge that it is working for evil. But I learned that this gentleman did not put in an appearance. Why? Echo answers, "Lack of moral courage." (This gentleman has one of the diplomas). I was made a permanent member of the State Society in its early life, but resigned on the introduction of this subject, as I could not countenance the proposition. Neither have I been modest in giving my views against the continuance of the work. Now, legions of dentists know *much* of the hollowness of the easy-getting degrees and in these days of such good omens for advance in the field of broader culture, being forced upon us by the demands of the times, does not good example cry out, *Down with all sham!* And let a degree come to mean that the bearer has pursued a legitimate course of study. And thus fraternal counsel will of itself come from all who stand on the basis that is characterized by broad culture.

If we are to have associations, of which none can become members but degreed men (which I do not personally object to), then I say, let honesty say this—their *insignia* means something more than outward embellishment, if not, hush about elevating the profession, this can only come from the *inside lever*.

I feel a sense of pity for those I have known so many years, and with whom, too, an affection has grown up that cannot be obliterated; to see their good names so tarnished.

I predict not many years will go by before some of these door-plates with D.D.S. (or M.D.) on them will be the passion of Peter Funk of the junk shop.

My opportunities have not been very limited in my insight of the "green room" of diploma business, and I would suggest moderation, but not fear, in attacking this matter of the New York State Society.

I do not wish it understood that I have commented on this subject for the sake of creating hot blood. I do it hoping the knife of criticism will cut this blemish from an organization that has done, and can do, much that is helpful—and more—remove a barrier so that all good men can conscientiously fraternize in its membership. Now, if those outside of New York think that all dentists in the State feel proud of this cheap diploma trade, don't be deluded for they do not.

A Close Observer.

